# Joint Brain Parametric T_1_-Map Segmentation and RF Inhomogeneity Calibration

**DOI:** 10.1155/2009/269525

**Published:** 2009-08-23

**Authors:** Ping-Feng Chen, R. Grant Steen, Anthony Yezzi, Hamid Krim

**Affiliations:** ^1^Department of Electrical and Computer Engineering, North Carolina State University, NC 27695, USA; ^2^Medical Communications Consultants, LLC 103 Van Doren Place Chapel Hill, NC 27517, USA; ^3^School of Electrical and Computer Engineering, Georgia Institute of Technology, GA 30332, USA

## Abstract

We propose a constrained version of Mumford and Shah's (1989) segmentation model with an information-theoretic point of view in order to devise a systematic procedure to segment brain magnetic resonance imaging (MRI) data for parametric T_1_-Map and T_1_-weighted images, in both 2-D and 3D settings. Incorporation of a tuning weight in particular adds a probabilistic flavor to our segmentation method, and makes the 3-tissue segmentation possible. Moreover, we proposed a novel method to jointly segment the T_1_-Map and calibrate RF Inhomogeneity (JSRIC). This method assumes the *average *T_1_ value of white matter is the same across transverse slices in the central brain region, and JSRIC is able to rectify the flip angles to generate calibrated T_1_-Maps. In order to generate an accurate T_1_-Map, the determination of optimal flip-angles and the registration of flip-angle images are examined. Our JSRIC method is validated on two human subjects in the 2D T_1_-Map modality and our segmentation method is validated by two public databases, BrainWeb and IBSR, of T_1_-weighted modality in the 3D setting.

## 1. Introduction

Brain structure segmentation is the apportionment of brain tissue into gray matter and white matter, based on the appearance of tissue in images produced by magnetic resonance imaging (MRI). Because manual tracing of the boundaries between tissues in the brain is labor intensive, difficult, error-prone, and unrealistic for large amounts of data, an automated or semiautomated segmentation technique is needed for either visualization or diagnosis. Different imaging modalities, such as T_1_-weighted, T_2_-weighted, or Proton Density (PD) images, have been used for different segmentation methods. T_1_-weighted images, because of their good contrast [1], have been widely tested for various segmentation methods [2–6]. A T_1_-Map is a parametric image of pure T_1_ (spin lattice relaxation time), derived from the solution of an equation describing tissue relaxation, and a parametric T_1_-Map which is different from a T_1_-weighted image. The relationship between T_1_ and several diseases, such as schizophrenia or sickle cell disease, has been studied [7–10], and T_1_ may be used as a possible indicator of pathology. Change in T_1_ of certain voxels in the brain over time may be an early indicator of possible pathology [8]. Therefore, the segmentation of a parametric brain T_1_-Map may highlight pathology unseen by other imaging approaches. 

 Past research has studied the segmentation of cortex in the brain. The three tissues white matter (WM), gray matter (GM), and cerebrospinal fluid (CSF) constitute the main parts in the brain. The goal is to find their respective boundaries. Different methods have been proposed to achieve this goal, and they may be classified into various categories: fuzzy segmentation methods [4, 11], Markov random field (MRF) methods [12, 13], Bayesian methods [14], active contour methods [1, 5, 6], or the combinations of two or more techniques. Some of these combinations are as follows: Leemput et al*.* [3] used expectation maximization (EM) and MRF, Xu et al*.* [2] used fuzzy segmentation and deformable surfaces, and Zhang et al*.* [15] combined a hidden Markov random field and an EM algorithm. Our method falls in the active contour category in the region-based formulation. It is an adaptive version of Mumford-Shah's model [16] to systematically segment different tissues in the brain. 

 Before the segmentation, several issues arise for obtaining a T_1_-Map. A T_1_-Map may be calculated by a rapid method known as the *variable nutation* method [17] which provides comparable precision but much faster speed over conventional methods [18]. This method requires the acquisition of a set of flip angle images and the T_1_ information can be extracted therein. The problem of determining the set of optimal flip angles therefore was studied. Deoni et al*.* [18, 19] proposed methods to determine the optimal flip angles by basically maximizing the signal-to-noise ratio (SNR) of a T_1_-Map. Their first work [18] required the knowledge of average TR/T_1_ in advance though, and their second work [19] introduced a weighted least square method to estimate the angles. We take another approach to determine the flip angles to achieve the trade-off between acquisition time and T_1_ accuracy. 

 Since a T_1_-Map is generated by a set of flip angle images, alignment of the images is important in order to obtain an accurate T_1_-Map. We therefore propose a method to register the raw data. The registration of flip angle images is usually ignored though because the movement of the head in the coil is minute. However, slight registration errors affect the resulting T_1_-Map dramatically, as will be shown. 

 Radiofrequency (RF)-inhomogeneity is another unavoidable problem encountered in MR imaging. The nonuniform distribution of the RF field can cause the resulting images to have low contrast and inhomogeneous intensity, which makes quantitative description and segmentation of the image difficult [20]. RF inhomogeneity affects the generation of T_1_-Map in the sense that the *spins* are not tilted by the predefined *nominal angles* [17, 20–22]. Hence, we focus on calibrating the RF nonuniformity in order to generate an accurate T_1_-Map. Cheng and wright [17] calculated an analytical form of T_1_ errors induced by RF nonuniformity and allowed simple correction of T_1_ measurements. Both Wang et al*.* [20] and Venkatesan et al*.* [22] incorporate a scaling factor *α* to rectify the nominal flip angles in their models. We propose a method, which assumes the *average* T_1_ value among a transverse plane is the same across the central brain slices, to jointly segment a T_1_-Map and calibrate the RF nonuniformity along the direction perpendicular to the transverse plane. This assumption may seem disputable because T_1_ indeed exhibits regional heterogeneity in human cortex [23]. Taking into account that we average out the heterogeneity of T_1_, and doing so only within few central brain slices, this assumption help calibrate RF-inhomogeneity and generate a quantitative T_1_-Map for segmentation. 

 The paper is organized as follows. In Section 2 we state our adapted model, with a fast implementation method. In Section 3 we first illustrate some necessary preprocessing to generate an accurate T_1_-Map, which includes the registration of flip angle images with the generation of a brain mask and the determination of optimal flip angles, to achieve a trade-off between quality and efficiency. We then describe a systematic procedure to segment a brain T_1_-Map. In Section 4 we propose a novel method to jointly segment a T_1_-Map and calibrate RF-inhomogeneity, and in Section 5 we show the results of registering the flip angle images, determining optimal flip angles, and segmenting the T_1_-Maps. We also show validations, the resulting T_1_-Map after RF-calibration, and the 3D segmentation for two sets of T_1_-weighted data. At last in Section 6 we discuss our findings and offer some concluding remarks in Section 7.

## 2. Proposed Model for Segmentation

Active contour methods comprise a popular segmentation technique in which an initialized contour is driven by a partial differential equation (PDE) to minimize an energy functional designed to attract the contour toward image edges. Active contour methods can be classified into two categories: edge-based [24–27, 27–34] and region-based [16, 35–44] methods. Edge-based methods examine the gradient information of the image, and stop the contour whenever the gradient is high. However, there are many situations when the edge is not clearly characterized by the gradient, and it has been shown that region-based methods outperform edge-based methods [35–37, 41, 43, 44]. By examining some statistics of the region inside and outside of the active contour, and optimizing the separation of these two statistics, we may achieve a better segmentation performance, thus making region-based methods more attractive. 

 Our proposed model is a modified Momford and Shah [16] functional, and falls in the region-based category. Two adaptations of Mumford-Shah's model constitute the novelty of our proposed technique. First is the incorporation of an information-theoretic view, which characterizes the statistical property of data. The second is a selective weighting, similar to the weight parameter in [35], which favors erring towards one tissue type or another, and thus make 3-tissue segmentation possible.

### 2.1. Adapted Mumford-Shah Functional

We use a modified Mumford-Shah [16] energy functional:

(1) $$E\left(f_{R_{\text{in}}},f_{R_{\text{out}}},\vec{C}\right)=\underset{i=\text{in},\text{out}}{\sum }\beta_{i}\int_{R_{i}}\left\{{\left(f_{R_{i}}-G(I)\right)}^{2}+\nu {\left\|\nabla f_{R_{i}}\right\|}^{2}\right\}dx+\alpha \oint_{\vec{C}}ds,$$

where *f*_*R*_*i*__ approximate a function *G*(·) applied of the image *I* for region *R*_*i*_, *i* = in or out. *f*_*R*_*i*__ is smooth within each region *R*_*i*_, but not across the boundaries; $\vec{C}$ denotes the region boundaries, which is the *contour* of interest in this paper, and 0 ≤ *β*_*i*_ ≤ 1 are weights such that *β*_in_ + *β*_out_ = 1. By minimizing this functional we obtain a function *f*_*R*_*i*__ which is faithful to the image (first term) and smooth within each region but not across the boundaries (second term), while penalizing excessive length of the boundaries (last term). 

 The first adaptation is the function *G*(·) applied on to image *I* instead of the image itself. It is motivated by the work of Unal et al*.* [45]. Specifically, an information-theoretic approach for maximizing a probabilistic disparity measure, Jensen-Shannon (JS) divergence, was proposed. A constructed function *G*(·) characterizes the property of the probability density function (PDF) of the image intensity such as skewness (*G*(*I*) = *I* ^3^), or kurtosis (*G*(*I*) = *I* ^4^), relative to a Gaussian [46]. A proper choice of *G*(·) will capture the statistical characteristics of the data and will hence achieve a good segmentation. 

**Figure 1:**
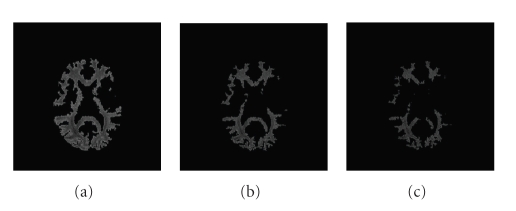
From left to right the weight *β*_in_ increases such that the segmented region becomes smaller, and therefore we obtain purer tissues.

 The second adaptation, similar to that in [35], is the selective weight *β*_*i*_ applied on the inside/outside energy terms. This provides a probabilistic assignment to the segmented regions, and also makes 3-tissue segmentation possible, as will be shown in Section 3. Enhancing the weight *β*_in_ is tantamount to penalizing both the error of the difference between the approximated function *f*_*R*_in__ and the data fidelity term *G*(*I*), as well as the degree of smoothness of *f*_*R*_in__. This would yield a smaller segmented region which is likely to be more faithful to the image, and of “purer” tissues. Figure 1 shows examples of white matter (WM) segmentation with different weights. With a larger inside-weight *β*_in_, the segmented region is smaller and the segmented tissues are purer.

### 2.2. Fast Mumford-Shah Implementation

Minimizing the Mumford-Shah energy functional involves solving for the approximating functions *f*_*R*_in/out__ and for the contour $\vec{C}$. The joint search for these infinite dimensional unknowns usually entails gradient descent flows. In particular, the approximated functions are typically modeled as a linear combination of a basis set whose dimension equals the number of pixels in the image, that is, each pixel is assumed to be independent [47]. The curve and the approximated functions are then evolved iteratively along the gradient flows. This implementation is computationally daunting and therefore a faster implementation method was in order. Alvino and Yezzi [48] proposed a fast implementation using a significantly smaller basis number to model the approximated functions, while still achieving sufficient resemblance to the obtained functions when using the pixel-by-pixel basis. 

 We adopt their so-called *linear heat equation basis* with the change from *I* to *G*(*I*) to incorporate the statistical properties of the data from an information-theoretic point of view in the previous section. We hence have,

(2) $$f_{R_{i}}=\gamma_{1,i}G\left(I\right)+\gamma_{2,i}\text{mean}\left(G\left(I\right)\right),$$

where mean(·) is the average function, and the coefficients *γ*_*j*,in(out)_, *j* = 1,2, may be similarly derived in [48]. Our derivations are included in the appendix. 

 Substituting (2) into (1), and using a classical methods of variational calculus, the gradient descent evolution of the curve may be derived as

(3) $$\begin{array}{cc} \frac{\partial \vec{C}}{\partial t} & =\{\beta_{\text{out}}\left[{\left(f_{R_{\text{out}}}-G(I)\right)}^{2}+\nu {\left\|\nabla f_{R_{\text{out}}}\right\|}^{2}\right]\\ & \;\;-\beta_{\text{in}}\left[{\left(f_{R_{\text{in}}}-G(I)\right)}^{2}+\nu {\left\|\nabla f_{R_{\text{in}}}\right\|}^{2}\right]\}N-\alpha \kappa N, \end{array}$$

(4) $$\begin{array}{cc} & =\left(\beta_{\text{out}}F_{\text{out}}-\beta_{\text{in}}F_{\text{in}}\right)N-\alpha \kappa N, \end{array}$$

where *κ* denotes the curvature of the contour $\vec{C}$, *t* an artificial time evolution parameter, and **N** the outward normal of the contour. *F*_in/out_ are derived by substituting *f*_*R*_in/out__ from (2) into (3) as shown in the appendix.

## 3. Segmentation of a Parametric Brain T_1_-Map

A brain parametric T_1_-Map is the calculated result (*variable nutation* method [17]) from several flip angle images. Because the flip angle images are acquired at different times, and because the subject may move during image acquisition, registration should be carried out first to obtain an accurate T_1_-Map. Moreover, in order to achieve a balance between the acquisition time and the resulting T_1_-Map quality, we propose a method to determine optimal flip angles. In the following subsections we will first illustrate how we register the flip angle images and obtain a brain mask as a byproduct, then describe our method to determine a set of optimal flip angles, and at last describe our proposed procedure to segment a T_1_-Map.

### 3.1. Registration of Flip-Angle Images and Generation of Brain Mask

The value of T_1_ is traditionally determined by acquisition methods such as Inversion Recovery (IR) or Saturation-Recovery (SR). Other rapid methods, such as *variable nutation* (the DESPOT method) have been proposed [17], and require acquisition of several flip angle images, and calculation of T_1_. Since these flip angle images are acquired at different times, registration must first be accomplished. Even though the interval between consecutive scans may be as short as two minutes, and movement of the subject's head inside the receiver coil may be a few pixels (under the resolution of a 256 × 256 image), the effects of such off-registration can be significant, as shown (Figure 5). Here we describe a method to register flip angle images and jointly obtain a mask, as a byproduct, to get rid of the skull and other structures around the brain. 

 We use a joint segmentation and registration (JSR) technique proposed by Yezzi et al*.* [49] with an additional tuning weight, to achieve registration and to obtain the mask. The theory of JSR technique consists of evolving two contours, with a enforced relationship (ex: rigid or affine transform) between them, in two images according to a partial differential equation (PDE) which is a result of optimizing, for example, the sum of two energy functionals. 

 For our particular task, we may choose a region-based energy, such as Chan and Vese's model [35] incorporating weights, which arises as a special case of (1) with the data term *G*(*I*) = *I* and the approximating function *f*_*R*_in/out__ = mean(*I*) inside and outside the contour. This model approximates the image *I* by a simple piecewise constant function, which suits our goal here because it creates a mask that divides the image into two parts- brain region and nonbrain region. 

 We observe that the boundary between the brain and nonbrain is visually more easily distinguished than the boundary between different tissues within the brain, for every flip angle image. We therefore put a very small weight *β*_in_ on the inside energy in (1) to penalize very little the difference between the data inside the contour, *I*, and the approximated function mean(*I*). Experimental results show that *β*_in_ = 0.4 yields satisfactory results. 

 We carry out this registration technique pairwise for all flip angle images, and once the flip-angel images are registered to each other, they are used to generate a T_1_-Map.

### 3.2. Determination of Optimal Flip-Angles

The determination of the flip angles that yields qualitative T_1_-Map and saves time, is mainly by comparing the difference between the gold standard and the T_1_-Maps generated from different combinatorial flip angles. We begin by acquiring images at a set of 19 flip angles which spans the range of *standard angles* [50] and will give an optimized T_1_-Map. This is also confirmed by observing that the generated T_1_-Map gives the best quality. We call this particular T_1_-Map the *gold standard*, or T_1_G__, and denote this set of angles as ${\vec{\theta }}_{19}$ = [2°, 5°, 10°, 15°, 20°, 25°, 30°, 35°, 40°, 45°, 50°, 55°, 60°, 65°, 70°, 75°, 80°, 85°, 90°]. We then compare the T_1_-Map generated from all combinations of the subset of ${\vec{\theta }}_{19}$ with T_1_G__. Out of these combinations we select the optimal subset of angles. Since the data acquisition is slice-based, this study of optimal flip angle focuses on the central slice, which is least affected by RF-inhomogeneity [51] and is routinely selected at the level of the basal ganglia, including both the genu and the splenium of the corpus callosum, and generally shows the putamen and lateral ventricle [52]. We denote the optimal *n*-angles ${\vec{\theta }}_{\text{opt},n}$, which is a subset of ${\vec{\theta }}_{19}$, as those that exhibit the smallest difference between T_1_G__ and the T_1_-Map generated by *n* flip angles:

(5) $${\vec{\theta }}_{\text{opt},n}=\arg\;\underset{{\vec{\theta }}_{n}}{\min\;}\underset{(x,y)\in \text{brain}}{\sum }\left|{\text{T}}_{1_{\text{G}}}\left(x,y\right)-T_{1_{{\vec{\theta }}_{n}}}\left(x,y\right)\right|,$$

where *n* ∈ {2,3,…, 19}, ${\vec{\theta }}_{n}\subset {\vec{\theta }}_{19}$, $T_{1_{{\vec{\theta }}_{n}}}$ denotes the T_1_-Map generated by ${\vec{\theta }}_{n}$, and the summation of (*x*, *y*) is over the whole brain region at the central slice. 

 Equation (5) therefore gives 18 sets, with the number of elements ranging from 2 to 19, of optimal angles. For the determination of the optimal set of flip angles, in reaching a compromise between efficiency and T_1_-Map quality, we examine the error between $T_{1_{{\vec{\theta }}_{\text{opt},n}}}$ and T_1_G__ and compute the error rate. The error rate is defined as

(6) $$E=\underset{(x,y)\in \text{brain}}{\sum }\frac{e\left(x,y\right)}{A\left(\text{brain}\right)}‍,$$

where *A*(brain) is the area of the brain, and *e*(*x*, *y*) is the error, defined as 1 if the difference between T_1_G__(*x*, *y*) and $T_{1_{{\vec{\theta }}_{\text{opt},n}}}(x,y)$ is greater than some threshold *ε*, and 0 otherwise. The threshold *ε* is defined as the minimum of the standard deviation among the T_1_ values of three brain tissues (WM, GM, and CSF) manually segmented by an expert. The summation of *e*(*x*, *y*) is over the whole brain at the central slice. 

 The plot of the error rate versus the number of flip angles is shown in Figure 6, where the inflection point of the fitted curve is at 10 flip angles (${\vec{\theta }}_{\text{opt},10}$ = [2°, 5°, 55°, 60°, 65°, 70°, 75°, 80°, 85°, 90°]). We hereafter routinely use these 10 flip angles to generate the T_1_-Map.

### 3.3. Brain T_1_-Map Segmentation Procedure

Once the brain mask is obtained (Section 3.1), it is used to segment away the skull, leaving only three major tissues in the image: WM, GM, and CSF. Notice that the curve evolution corresponding to the energy introduced in Section 2 always results in a “binary segmentation”, where we will have regions *inside* (foreground) and *outside* (background) the contour(s). We cannot simultaneously segment the three tissues, even though we will later talk about multiphase segmentation. We may, however, tune the weights (Section 2.1), to penalize the error between the data term and the approximated function (1) to segment one tissue at a time, with a progression analogous to that of “peeling an onion”. 

 We illustrate our T_1_-Map segmentation procedure for a *hard segmentation* of three tissues, and the probabilistic segmentation is obtained by varying the weights around the value of the trained weight (Section 5.4). This procedure is applicable to both 2D and 3D data sets. 

 A T_1_-Map segmentation procedure consists of three steps, where the first two steps are to evolve the contours by minimizing the energy in (1) with different weights, and the third step is just a simple subtraction. The procedure is as follows:

1. Treat WM as the foreground, everything else as the background; let *β*_in_ in (1) be the trained *β*_in,WM_, and use it to segment WM in the interior region of the contour.
2. Treat WM and GM as the foreground, CSF and everything else as the background; let *β*_in_ in (1) be the trained *β*_in,CSF_, and use it to obtain CSF in the exterior region of the curve filtered by the brain mask.
3. GM is obtained by subtracting WM and CSF from the whole brain.

The procedure is based on the anatomical observation that GM is enclosed by CSF, and that CSF is separated from WM [1, 3], such that we may peel off one layer at a time. 

 The choice of function *G*(*I*) in (1), which is chosen to better capture the statistical property of T_1_-Map (and for other modality), will be shown in Section 5. The values of *β*_in,WM_ and *β*_in,CSF_ are determined through a training process. Suppose an expert's manual segmentation is regarded as the ground truth. If *R*_*e*_ denotes the segmentation region by the expert, and *R*_*β*_in__ denotes the segmentation region by the weight *β*_in_ with some fixed *G*(*I*), for some tissue, then the value of *β*_in,tissue_ is determined by minimizing

(7) $$\beta_{\text{in},\text{tissue}}=\arg\underset{\beta_{\text{in}}}{\min\;}\left\{1-\frac{\left|R_{e}\cap R_{\beta_{\text{in}}}\right|}{\left|R_{\beta_{\text{in}}}\right|+\left|R_{e}\right|-\left|R_{e}\cap R_{\beta_{\text{in}}}\right|}\right\},$$

where |*R*| denotes the number of pixels in region *R*. The first term inside the bracket is an *overlap metric* (OM) [5] which will be discussed again in Section 4. It is commonly used in validating segmentation performance—the closer it is to 1, the better it has performed.

## 4. Joint T_1_-Map Segmentation and RF-Inhomogeneity Calibration

RF-inhomogeneity is an unavoidable problem in MR imaging. The strength of the RF field varies within the MR scanner, such that the resulting image may be of low contrast or of nonuniform intensity. In what follows, we will first describe how a T_1_-Map is calculated from a set of flip angle images, then how the T_1_-Map is affected by RF-inhomogeneity and then show our proposed correction method. 

### 4.1. Variable Nutation Method with RF-Inhomogeneity

A T_1_-Map is calculated by *variable nutation* [17]. Flip-angle images are acquired using a FLASH sequence at different flip angles, and T_1_ is calculated from the slope of a least square fitted line to the pair of data [*s*(*θ*)/sin *θ*, *s*(*θ*)/tan *θ*], where *s*(*θ*) is the signal strength of the flip angle image expressed as a function of the flip angle *θ*. 

**Figure 2:**
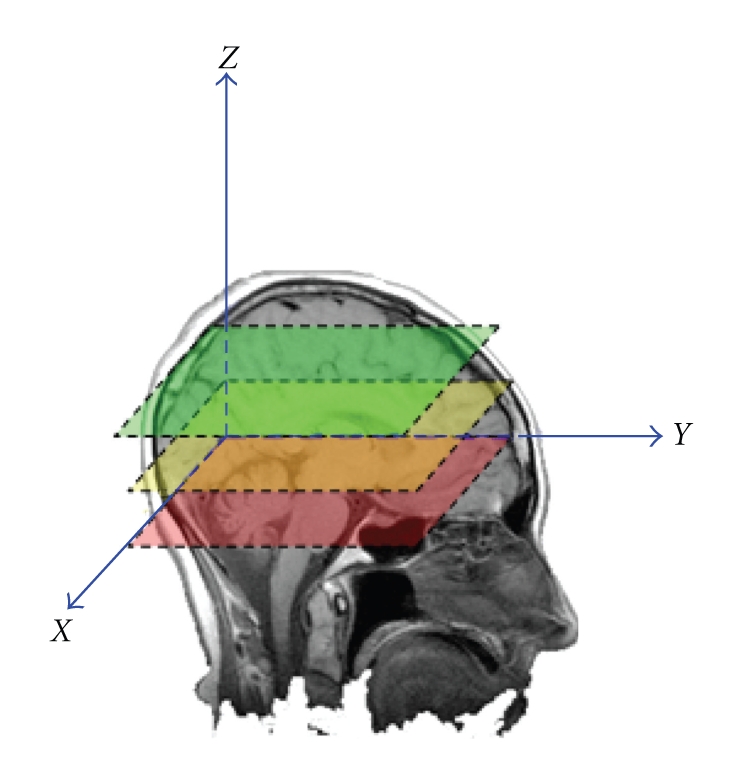
A carton illustrating the scanning orientation.

 RF-inhomogeneity affects the computation of T_1_ in that the spins are not tilted by the nominal angle because the strength of the RF field is not as predefined. This phenomenon appears more serious at the periphery of the receiver coil. Calibration of RF-inhomogeneity, or, the correction of flip angles, produces a more accurate T_1_-Map. A spatially dependent scaling factor *α*, therefore, is introduced to adjust the tilted angle [20, 22], that is, replacing *θ* by *αθ*

(8) $$\left[\frac{s\left(\alpha \theta \right)}{\sin\left(\alpha \theta \right)},\frac{s\left(\alpha \theta \right)}{\tan\left(\alpha \theta \right)}\right],$$

and T_1_ is extracted from the slope of the line fitted to the above space. Our imaging setting is slice-based across different transverse planes (Figure 2), thus our proposed joint segmentation and RF-inhomogeneity calibration (JSRIC) method is as an initial step to easily calibrate RF-inhomogeneity vertically.

### 4.2. Flip Angle Rectification and Segmentation of T_1_-Maps

Our method is based on the assumption that the *average* T_1_ of WM should be roughly the same across central brain transverse slices. T_1_ exhibits regional heterogeneity in human cortex [23]. We however average out the heterogeneity of T_1_, and carry out the method only within few central brain slices. Our method therefore yields a better T_1_-Map for segmentation and jointly rectifies flip angles. The advantage is that as the segmentation delineates more precisely the WM region, flip angle correction is therefore enhanced, and as flip angle correction is carried out more precisely, which gives better quality T_1_-Map, segmentation is facilitated as well. 

 Our joint segmentation and RF-inhomogeneity calibration (JSRIC) method requires first taking the average T_1_ value for segmented WM at the central slice (which has relatively uniform B1 field [51, 53]) as the reference, and then iteratively segmenting and searching for the scaling factor *α* in (8) for all other slices. This is a three-step iterative process (as shown inside the dashed box in flowchart 3).

1. Segment WM for the *central slice* by the method proposed in Section 3.3, compute the average T_1_ value of WM, and denote the average as *M*.
2. For slice *m*, varies *α* in (8), and calculate the corresponding T_1_-Map, denoted as T_1_(*α*). If *α* goes beyond *α*_max_, claim the current slice as uncalibratable and repeat this step for the next slice *m* + 1.
3. Segment WM for T_1_(*α*), compute the average T_1_ of WM, and denote it by *L*(*α*). If *M* ≠ *L*(*α*), go back to step 2 and increase *α*, otherwise claim it is done for the current slice.
4. Iterate steps 2 and 3 through all the slices.

The variables *α*_min_, *α*_max_, and Δ*α* in flowchart 3q are all predefined parameters.

## 5. Experimental Results

In this section we show a series of results from segmenting a parametric brain T_1_-Map, which includes the registration of flip angle images, generation of a brain mask as a byproduct, determination of the optimal flip angles, and joint T_1_-Map segmentation and RF-inhomogeneity calibration. In addition, we will apply our proposed segmentation method to another modality, T_1_-weighted images, in a 3D setting, to show the generality of our segmentation method. Results for both modalities (T_1_-Map and T_1_-weighted) will be validated.

### 5.1. Subjects and Scan Protocol

The subjects being scanned were recruited from a clinic in the Department of Psychiatry at the University of North Carolina, under an IRB-approved protocol to image the brain. Informed consents were signed by subjects or their guardians. A transverse 3D FLASH sequence using different flip angles was acquired in a 3T Siemens MRI scanner with a quadrature head coil. The scan parameters were: TR = 25 msec, TE = 2.83 msec, 16 slices, and slice thickness = 5 mm. The center slice used for optimal flip angle study was routinely selected as described (Section 3.2).

### 5.2. Registration of Flip-Angle Image and Brain Mask

The registration of a set of flip angle images is carried out pair wise, and Figure 4 shows two flip angle images and the resulting brain mask. This mask has otherwise to be generated manually or by other techniques [54]. Note that even though the flip angle images are off-registered by no more than four pixels in both the *x*- and *y*-directions, the impact is obvious. Figure 5 shows two T_1_-Maps generated by registered and unregistered flip angle images, and we can see that after registration the anatomical structure of the T_1_-Map is more readily distinguished, and the high signal intensity artifact in the upper left of the unregistered T_1_-Map map is reduced.

### 5.3. Determination of Optimal Flip-Angles

The plot of the error rate *E* (defined in (6)) versus the number of flip angle is shown in Figure 6, and Section 3.2 already concluded that a set of 10 flip angles (${\vec{\theta }}_{\text{opt},10}$ = [2°, 5°, 55°, 60°, 65°, 70°, 75°, 80°, 85°, 90°]) is a good compromise between T_1_ quality and efficiency. Figure 7 shows a comparison of generated T_1_-Maps when using 2 optimized flip angles ([5°, 55°]), 6 optimized flip angles ([2°, 5°, 60°, 65°, 70°, 75°]) and 10 optimized flip angles, with an “error map” (error defined in Section 3.2) for each image. The T_1_-Map generated by 2 angles exhibits substantially more error than the maps using 6 or 10 angles, as shown by the bright pixels in the error map. We conclude that 10 flip angles is an acceptable choice considering the trade-off between accuracy and scan time. 

### 5.4. Flip-Angle Rectification and Segmentation of T_1_-Maps

**Figure 3:**
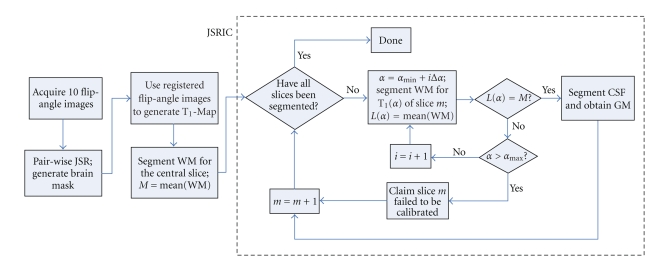
A flowchart for the whole procedure to jointly segment T_1_-Map and calibrate RF-inhomogeneity. The procedure includes the registration of flip angle images, brain mask generation, and our JSRIC method.

**Figure 4:**
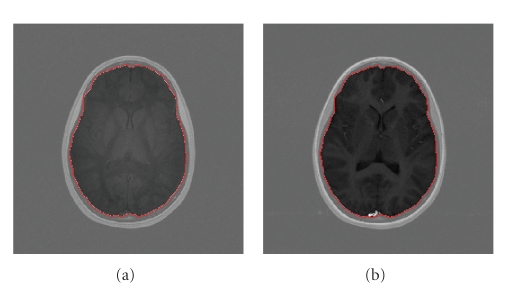
JSR technique: two active contours evolve in two flip angle images (of angles 5° and 40°) to jointly register and segment (generate the brain mask) the brain. The region outside the mask are brightened to better show the contrast.

**Figure 5:**
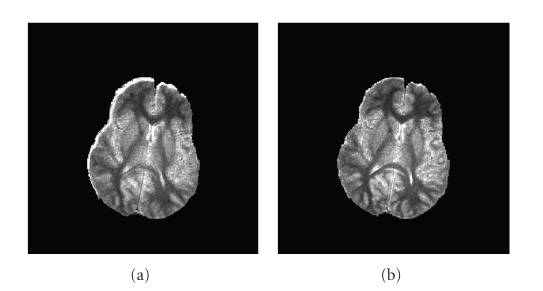
The T_1_-Map generated by (a) unregistered and (b) registered flip angle images. It can be seen that the anatomical structure of the registered one is clearer than the other.

**Figure 6:**
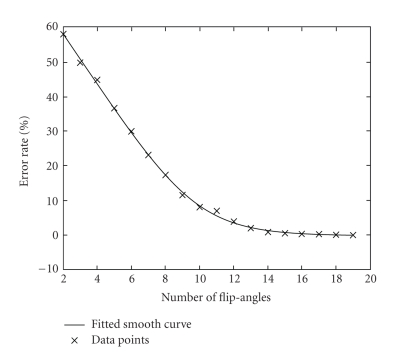
The error rate *E* versus number of flip angles, and 10 flip angles is at the inflection point of the fitted curve.

**Figure 7:**
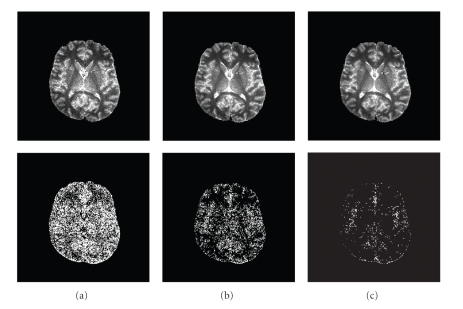
The T_1_-Maps generated by (a) 2 (b) 6 and (c) 10 flip angles on the top row, and their corresponding error maps on the second row.

We carried out JSRIC introduced in Section 4 on the T_1_-Maps of two subjects. Out of a total of 16 slices, the bottom 4 slices were discarded because they did not cover sufficient WM for segmentation by our JSRIC method. The method follows the flowchart in the dashed box shown in Figure 3 from slice 5 to slice 15. Slice 16 could not be calculated, possibly because of its proximity to the boundary of the RF field and hence degraded. 

**Figure 8:**
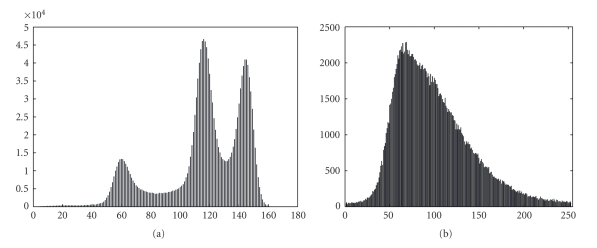
Histogram of a (a) T_1_-weighted image and a (b) T_1_-Map.

 The function *G*(*I*) introduced in (1) was empirically chosen as the cubic function *I* ^3^, which characterizes the *skewness* of a PDF. Moreover, *β*_in,WM_ and *β*_in,CSF_ are obtained by training according to (7) based on an expert's manual segmentation of one subject (*training subject*). These values are *β*_in,WM_ = 0.93 and *β*_in,CSF_ = 0.53. The same values are then applied to the other subject (*testing subject*). The segmentation of a T_1_-Map is achieved by evolving contours according to (4). Since the curve evolution is based on a gradient flow, the final result may vary depending upon the initialization. A good initialization may avoid an undesirable local minimum. A T_1_-Map does not necessarily have good contrast compared to a T_1_-weighted image. A T_1_-weighted image histogram is shown (Figure 8(a)), and a spectral analysis can be carried out to threshold the image as an initialization [5, 55], to achieve a good segmentation after fine tuning of the contour. The T_1_-Map does not have the same level of contrast as a T_1_-weighted image (Figure 8(b)). We therefore initialize the contours by either placing uniformly spaced squares or by manually seeding (by mouse clicking and dragging on the image). Both methods give similar performance with manual seeding slightly better. We will therefore show the results with manual seeding for T_1_-Map.

**Figure 9:**
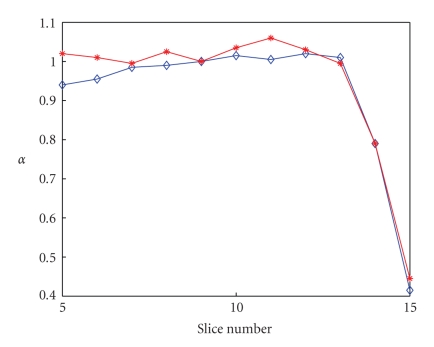
“*α* map”: The plot of *α* value versus slice number for two subjects.

**Figure 10:**
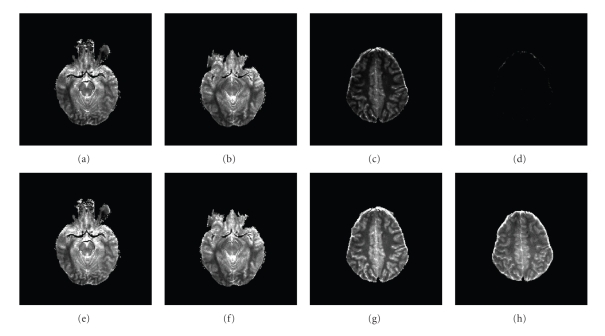
T_1_-Maps generated from unrectified (First row) and rectified (second row) flip angle images at slice number 5, 6, 14 and 15.

Figure 9 shows the “*α*-map” (*α* versus slice number) for two subjects, where the parameter *α* is as defined (Section 4.1). The strength of the RF field drops significantly at the top slices (slice 14 and 15) such that the corresponding flip angles have to be rectified by a scaling factor much smaller than 1. Figure 10 shows two T_1_-Maps generated with and without flip angle rectification for the same subject at slice number 5, 6, 14 and 15. It is clearly seen that the top 2 slices of the T_1_-Maps with calibration have much better quality than those without.

**Figure 11:**
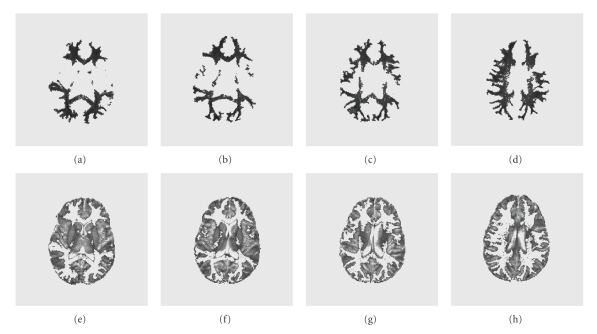
Segmentation result of WM (first row) and GM (second row) for the testing subject at slice number 10–13.

**Figure 12:**
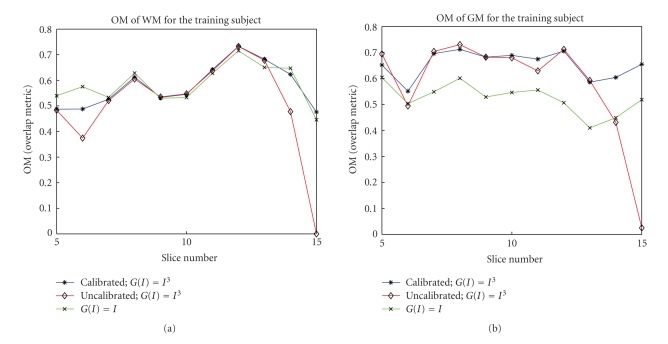
Overlap metric of (a) WM and (b) GM segmentations for calibrated and uncalibrated T_1_-Maps with cubic function *G*(*I*) = *I* ^3^ and tuned weights and also for calibrated T_1_-Map with function *G*(*I*) = *I* with tuned weights.

Figure 11 shows some selective segmentation results of WM and GM for the test subject. Validation of these results requires comparison with manually segmented images. The commonly examined metrics which determine the performance of a segmentation are TP (true positive), FP (false positive), and OM (overlap metric) [1, 5]. The overlap metric is defined for a class assignment as the sum of the number of pixels that both have the class assignment in each segmentation divided by the sum of pixels where either segmentation has the class assignment. This metric approaches 1 for segmentations that are very similar, and is near 0 when they share no similarly classified pixels. The OM metric is usually compared for different segmentation methods. Figure 12 shows three overlap metric curves (OM versus slice number) of WM and GM segmentation for the training subject (the test subject exhibits a similar result). The first curve corresponds to the segmentation of calibrated T_1_-Maps, with our tuned weights and cubic function *G*(*I*) = *I* ^3^, the second corresponds to the T_1_-Maps generated by nonrectified flip angles (using the same segmentation parameters), and the third corresponds to the calibrated T_1_-Map, with function *G*(*I*) = *I* and tuned weights (*β*_in,WM_ = 0.9 and *β*_in,CSF_ = 0.7). These results show that calibrated T_1_-Maps enhance the segmentation performance compared to un-calibrated T_1_-Maps, especially at the top two slices (slice 14 and 15) which are most seriously affected by RF-inhomogeneity. The comparison of different functions *G*(*I*) shows that the performance of WM segmentation is comparable for the two functions. There is, however, a significant difference for CSF segmentation, thus affecting GM segmentation. The cubic function *I* ^3^ outperforms *I* tremendously for GM segmentation. It also demonstrates that the cubic function better characterizes the statistical properties, the skewness, of the data. The OM for the other subject also has similar results.

**Figure 13:**
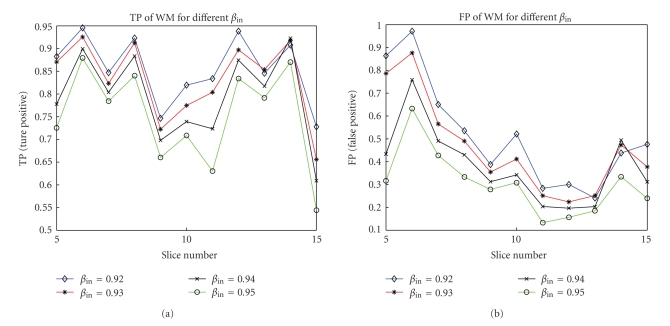
(a) TP (True Positive) and (b) FP (False Positive) of WM segmentation for different weights *β*_in_.

Figure 13 shows TP and FP for WM segmentation with different weights *β*_in_ around the value of *β*_in,WM_, to demonstrate the notion of our probabilistic segmentation. TP = |*R*_*β*_in__∩*R*_*e*_|/|*R*_*e*_|, and FP = (|*R*_*β*_in__| − |*R*_*β*_in__∩*R*_*e*_|)/|*R*_*e*_|, where *R*_*e*_ and *R*_*β*_in__ are the expert segmented regions and ours using *β*_in_ respectively, and |*R*| denotes the area of region *R*. When the weight increases, so does the penalty for the error between the data term and the approximating function (1). Therefore TP and FP decrease correspondingly, and vice versa.

### 5.5. Segmentation of T_1_-Weighted Images

In this section we show the generality of our proposed segmentation method in a 3D setting by applying it to a commonly exploited modality—T_1_-weighted images. The same procedures are carried out as in Section 3.3, except that now the images are collated into volumes and the active contour is replaced by an *active surface*. We test it on two open databases accessible online— BrainWeb (http://www.bic.mni.mcgill.ca/brainweb/) [56] and IBSR (http://www.cma.mgh.harvard.edu/ibsr/). The former is a simulated brain MRI database, therefore ground truth is provided and the latter is genuine brain MRI data which also has been manually segmented by experts. We preprocessed the data to filter out everything except for the three main tissues, WM, GM, and CSF. 

**Figure 14:**
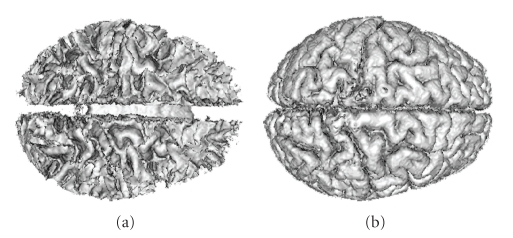
3D segmentation results of (a) WM and (b) GM for the testing simulated brain.

 We first tested our segmentation method on two BrainWeb subjects (a training and a testing subject) using simulated brain T_1_-weighted data. The images are 1 mm slice thick, with 3% noise level, and 20% RF intensity nonuniformity (INU) and each data set was 217 × 181 × 106 pixels. Because the contrast between different tissues was high, we did a histogram analysis and applied the threshold method similar to [5] for initialization. The function *G*(*I*) is still chosen as *I* ^3^, and *β*_in,WM_ and *β*_in,CSF_ are obtained by training as 0.3 and 0.2 respectively. The validation metrics for the testing subject are shown in Table 1. The results show that it achieves a high performance of OM being around 0.8 for three tissues. The computational time for one subject is less then 1 minute on a laptop with a 1.73 GHz CPU and 1 GB memory. Figure 14 shows the segmentation results for the testing brain. 

**Figure 15:**
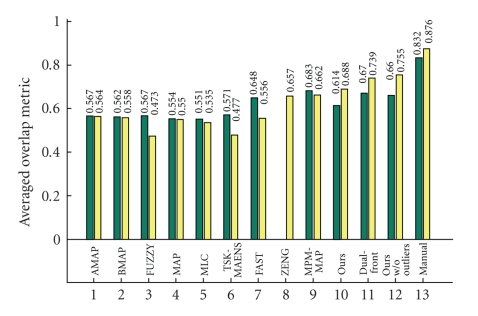
Average OM for WM and GM segmentations on 20 normal real brain data of T_1_-weighted modality. The left and right columns denote the average OM of WM and GM segmentation respectively. Some statistics are from IBSR and others are from [5]. They represent: AMAP: adpaptive MAP, BMAP: biased MAP, FUZZY: fuzzy C-means; MAP: Maximum a posteriori probability, MLC: maximum likelihood, TSK-MEANS: tree-structure *k*-means, FAST: hidden Markov method [15], ZENG: coupled-surface method [1], MPM-MAP: Bayesian method [14],and Dual-front: Dual-front method [5].

**Figure 16:**
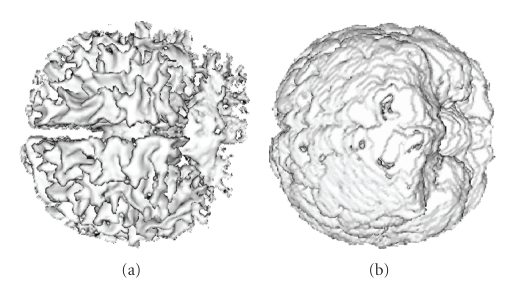
3D segmentation results of (a) WM and (b) GM for one real brain data of T_1_-weighted modality.

 We then tested our segmentation method on 20 data sets of T_1_-weighted images provided by the Center for Morphometric Analysis at Massachusetts General Hospital on the IBSR website. The data set for each subject was 256 × 256 × *l*, where *l* ranges from 58 to 63 pixels. We arbitrarily chose one subject (subject 1_24) as the training data, empirically chose the function *G*(*I*) = *I* ^3^ for WM and *G*(*I*) = *I* for CSF, *f*_*R*_in(out)__ = mean_in(out)_(*G*(*I*)), *β*_in,WM_ = 0.75, and *β*_in,CSF_ = 0.2. We did not carry out any preprocessing to denoise the data or to decrease the RF-inhomogeneity effect which seriously degraded the data. Therefore the spectral analysis could not be carried out and we used uniformly spaced cubes as the initialization. Out of 20 subjects we however still have 3 particularly unsuccessful cases (specifically subject 2_4, 16_3, and 111_2) due to RF-inhomogeneity which we did not rectify. Including these three subjects, we have an *averaged overlap metric* around 0.651, and if excluding these 3 outliers, we achieved an averaged OM of around 0.707. The computational time is less then 5 minutes on the same PC as above. Figure 15 shows the OM for WM and GM segmentations compared to other techniques. The statistics show that our method outperforms most other methods even if the outliers are included; if excluding those, we have WM segmentation slightly poorer but GM segmentation slightly better than the current best segmentation method. Figure 16 shows the 3D segmentation for one subject.

## 6. Discussion

### 6.1. Registration of Flip-Angle Images and Generation of Brain Mask

To register flip angle images we used the JSR technique, which has the advantage of jointly segmenting, registering, and generating a brain mask. Other techniques such as the information-theoretic method [57] only register the images, and the brain mask has to be otherwise generated. Our JSR has been carried out pair wise on 10 flip angle images, registering 9 images to a reference image. In theory, however, it should be possible to integrate multiple-image in JSR's formulation. By summing the energy functionals of multiple images with a relationship enforced between each contour, that is, *E*(*C*_1_, *g*_2_,…, *g*_*n*_) = *E*_1_(*C*_1_) + *E*_2_(*g*_2_(*C*_1_)) + ⋯ + *E*_*n*_(*g*_*n*_(*C*_1_)), where *C*_*i*_ = *g*_*i*_(*C*_1_), *i* = 1,…, *n*, the evolution of the contours *C*_*i*_ and registration parameters *g*_*i*_ may be derived similarly.

### 6.2. Segmentation of Brain Data and Probabilistic Segmentation

Our segmentation of three brain tissues is based on the tuning of weights, to penalize differently the error of the approximated function, to obtain different regions of tissue. Segmentation uses the anatomical nature of brain tissue which has a layered structure such that we may peel off one layer at a time. Our method uses an active contour that is able to separate an image into two parts: the inside and the outside of the contour. However, *multiphase active contour* techniques exist [36, 58] and are able to evolve multiple contours simultaneously and segment multiple regions at once. These methods should theoretically be able to segment three brain tissues simultaneously. Our method is different in the sense that it has a probabilistic flavor that the tuning weights determine a *purity-level* of segmented tissues.

### 6.3. Joint Segmentation and RF-Inhomogeneity Calibration

Our JSRIC method works by enforcing the average of white matter T_1_ value to be homogeneous across different transverse planes in the central brain region, to find the scaling factors *α* which affects the flip angles. Even though T_1_ has regional heterogeneity, by taking the average of WM for the transverse plane we average out this heterogeneity, and doing so only in the central brain region. WM segmentation and RF-calibration enhance the precision of one another, as shown in the performance plot in the previous section.

## 7. Conclusion

In conclusion, we propose an adapted Mumford-Shah type energy functional for segmentation. The two adaptations are: (1) a function *G*(*I*) is able to characterize the statistical properties of the data to achieve better segmentation results, and (2) the tuning weights *β*_in(out)_ are able to segment brain tissues in a probabilistic fashion and achieve three-tissue segmentation. We also propose a novel method (JSRIC) to jointly segment a T_1_-Map and calibrate RF-inhomogeneity. The whole procedure moreover includes the determination of optimal flip angles in achieving the balance between accuracy and efficiency, and joint registration of flip angle images and generation of brain mask. After RF-calibration, the top and bottom slices of T_1_-Maps show better contrast and enhance the segmentation performance. The segmentation method has also been applied to T_1_-weighted data, to show the generality of our segmentation method, and the results are validated by ground truth and by expert manual segmentations. The results show high performance with our method.

## Figures and Tables

**Table 1:** Different validation metrics for a simulated brain data of T_1_-weighted modality.

	WM	GM	CSF
TP(%)	89.8	91.8	85.1
FP(%)	6.9	12.5	9.6
OM	0.84	0.817	0.776

## Appendix

### Derivation of Gradient Flows for Our Proposed Energy

To derive the gradient flow of our energy in (1) with Alvino et al*.*'s [48] fast Mumford-Shah implementation, we first restate their result for the approximated function in scale space. Suppose the approximated function *f*_*R*_*i*__, *i* = in or out, is expressed as a linear combination of basis Φ_*j*_:

(A.1) $$f_{R_{i}}=\overset{N}{\underset{j=1}{\sum }}\gamma_{j,i}\Phi_{j},$$

then the coefficients *γ*_*j*,*i*_ can be solved by a linear system

(A.2) $$\Gamma_{i}+\Lambda_{i}{\vec{\gamma }}_{i}=\Xi_{i},$$

where

(A.3) $$\begin{array}{c} \Gamma_{i}=\left[\begin{array}{ccc} \int_{R_{i}}^{}\Phi_{1}\Phi_{1} & \cdots & \int_{R_{i}}\Phi_{N}\Phi_{1}\\ \int_{R_{i}}\Phi_{1}\Phi_{2} & \cdots & \int_{R_{i}}\Phi_{N}\Phi_{2}\\ \vdots & & \vdots \\ \int_{R_{i}}\Phi_{1}\Phi_{N} & \cdots & \int_{R_{i}}\Phi_{N}\Phi_{N} \end{array}\right],\\ \Lambda_{i}=\left[\begin{array}{ccc} \int_{R_{i}}\langle \nabla \Phi_{1},\nabla \Phi_{1}\rangle & \cdots & \int_{R_{i}}\langle \nabla \Phi_{N},\nabla \Phi_{1}\rangle \\ \int_{R_{i}}\langle \nabla \Phi_{1},\nabla \Phi_{2}\rangle & \cdots & \int_{R_{i}}\langle \nabla \Phi_{N},\nabla \Phi_{2}\rangle \\ \vdots & & \vdots \\ \int_{R_{i}}\langle \nabla \Phi_{1},\nabla \Phi_{N}\rangle & \cdots & \int_{R_{i}}\langle \nabla \Phi_{N},\nabla \Phi_{N}\rangle \end{array}\right], \end{array}$$

and each *N* × *N* matrices must be computed for each region. Also,

(A.4) $${\vec{\gamma }}_{i}=\left[\begin{array}{c} \gamma_{1,i}\\ \gamma_{2,i}\\ \vdots \\ \gamma_{N,i} \end{array}\right],\;\;\Xi_{i}=\left[\begin{array}{c} \int_{R_{i}}\Phi_{1}G\left(I\right)\\ \vdots \\ \int_{R_{i}}\Phi_{N}G\left(I\right) \end{array}\right]$$

are each *N* × 1 vectors.

Therefore we may replace the basis Φ_*j*_ by *G*(*I*) and mean(*G*(*I*)) as in (2), and solve for the coefficients ${\vec{\gamma }}_{i}$:

(A.5) $$\gamma_{1i}=\frac{T_{i}-m_{i}S_{i}}{T_{i}+W_{i}-m_{i}S_{i}},\;\;\gamma_{2i}=\frac{W_{i}}{T_{i}+W_{i}-m_{i}S_{i}}$$

where

(A.6) $$\begin{array}{c} S_{i}=\int_{R_{i}}G\left(I\right)dxdy,\;\;m_{i}=\frac{S_{i}}{\int_{R_{i}}dxdy},\\ T_{i}=\int_{R_{i}}G{\left(I\right)}^{2}dxdy,\;\;W_{i}=\int_{R_{i}}{\left\|\nabla G(I)\right\|}^{2}dxdy. \end{array}$$

Then substituting the coefficients in (2) by the above results and then pluging *f*_*R*_in(out)__ into (3), we have

(A.7) $$\begin{array}{ll} F_{\text{out}} & ={\left[(\gamma_{1,\text{out}})G(I)+\gamma_{2,\text{out}}m_{\text{out}}\right]}^{2}+{\left[\gamma_{1,\text{out}}\left\|\nabla G(I)\right\|\right]}^{2},\\ F_{\text{in}} & ={\left[(\gamma_{1,\text{in}})G(I)+\gamma_{2,\text{in}}m_{\text{in}}\right]}^{2}+{\left[\gamma_{1,\text{in}}\left\|\nabla G(I)\right\|\right]}^{2}, \end{array}$$

in (4).
